# Effects and action mechanisms of individual cytokines contained in PRP on osteoarthritis

**DOI:** 10.1186/s13018-023-04119-3

**Published:** 2023-09-22

**Authors:** Zhengchao Wang, Pengfei Zhu, Bokai Liao, Hongbo You, Yu Cai

**Affiliations:** 1 Department of Orthopedics, Wuhan Fourth Hospital, Wuhan, China; 2 Department of Cardiovascular, Wuhan Fourth Hospital, Wuhan, China; 3 School of Chemistry and Chemical Engineering, Guangzhou University, Guangzhou, China; 4 Department of Orthopedics, Tongji Hospital, Tongji Medical College, Huazhong University and Technology, Jiefang Avenue No.1095, Qiaokou District, Wuhan, 430030 Hubei Province China; 5 Department of Rehabilitation, Wuhan Fourth Hospital, Hanzheng Street No.473, Qiaokou District, Wuhan, 430000 Hubei Province China; 6 Department of Cardiovascular, Fujian Medical University Union Hospital, Fuzhou, China

**Keywords:** Platelet-rich plasma, Osteoarthritis, Cytokines

## Abstract

Osteoarthritis (OA) is defined as a degenerative joint disease that can affect all tissues of the joint, including the articular cartilage, subchondral bone, ligaments capsule, and synovial membrane. The conventional nonoperative treatments are ineffective for cartilage repair and induce only symptomatic relief. Platelet-rich plasma (PRP) is a platelet concentrate derived from autologous whole blood with a high concentration of platelets, which can exert anti-inflammatory and regenerative effects by releasing multiple growth factors and cytokines. Recent studies have shown that PRP exhibits clinical benefits in patients with OA. However, high operational and equipment requirements greatly limit the application of PRP to OA treatment. Past studies have indicated that high-concentration PRP growth factors and cytokines may be applied as a commercial replacement for PRP. We reviewed the relevant articles to summarize the feasibility and mechanisms of PRP-based growth factors in OA. The available evidence suggests that transforming growth factor-α and β, platelet-derived growth factors, epidermal growth factor, insulin-like growth factor-1, and connective tissue growth factors might benefit OA, while vascular endothelial growth factor, tumor necrosis factor-α, angiopoietin-1, and stromal cell derived factor-1α might induce negative effects on OA. The effects of fibroblast growth factor, hepatocyte growth factor, platelet factor 4, and keratinocyte growth factor on OA remain uncertain. Thus, it can be concluded that not all cytokines released by PRP are beneficial, although the therapeutic action of PRP has a valuable potential to improve.

## Background

Osteoarthritis (OA) is a degenerative joint disease characterized by cartilage degradation, osteophyte formation, and synovial inflammation [1]. This disease can affect all tissues in the joint, including the articular cartilage, subchondral bone, the ligament capsule, and the synovial membrane, leading to joint failure [2]. The conventional nonsurgical treatments for OA, such as hyaluronan, corticosteroids, nonsteroidal anti-inflammatory drugs, IL-4 and IL-1 receptor antagonists, paracetamol, and chondroitin sulfate products, are mainly focused on relieving pain and improving joint functions [3–6]; these treatments are ineffective for cartilage repair and induce only symptomatic relief. An increasing number of novel treatments have been developed recently, including platelet-rich plasma (PRP), vitamin D, oral collagens, methylsulfonylmethane, and curcumin [7–10]. However, not all of these treatments have relatively credible guidelines.

PRP is a platelet concentrate derived from autologous whole blood, that has a high concentration of platelets, containing 3–6 fold platelets compared to that in whole blood [11–13]. PRP exerts anti-inflammatory and regenerative effects, which are mediated through the release of multiple growth factors and cytokines, including platelet-derived growth factors (PDGF), transforming growth factor (TGF), vascular endothelial growth factor (VEGF), epidermal growth factor (EGF), fibroblast growth factor (FGF), connective tissue growth factor (CTGF), insulin-like growth factor (IGF), hepatocyte growth factor (HGF), keratinocyte growth factor (KGF), angiopoietin-1 (Ang-1), platelet factor 4 (PF4), stromal cell derived factor (SDF), and tumor necrosis factor (TNF) [14]. Currently, studies have demonstrated that PRP exhibits clinical benefits in patients with musculoskeletal disorders [15–17]. Moreover, systematic reviews and meta-analyses have concluded that OA patients can benefit from intra-articular administration of PRP [18–20]. Although PRP has been widely used in OA due to its regenerative potential, its efficacy remains debatable due to individual differences and different preparations, which lead to varied therapeutic effects. In particular, the different preparations of PRP may account for different effects on treating diseases in different studies.

As described previously, the high demand of operation and equipment limits the application of PRP [21]. If a patient receives PRP treatment, the patient’s blood is first drawn and centrifuged to collect the platelets. As a result, PRP is difficult to repeatedly administer and develop as a commercial drug for easy application. However, studies have indicated that high concentrations of growth factors and cytokines in PRP may be used as a commercial replacement for PRP [21, 22]. Many PRP-based growth factors and platelet cytokines have been examined in cells through in vitro and in vivo animal experiments to determine their effects and mechanisms in OA. We reviewed published articles and summarized the feasibility and mechanisms of PRP-based growth factors in OA.

## Cytokines that are beneficial for OA treatment (Fig. 1)

**Fig. 1:**
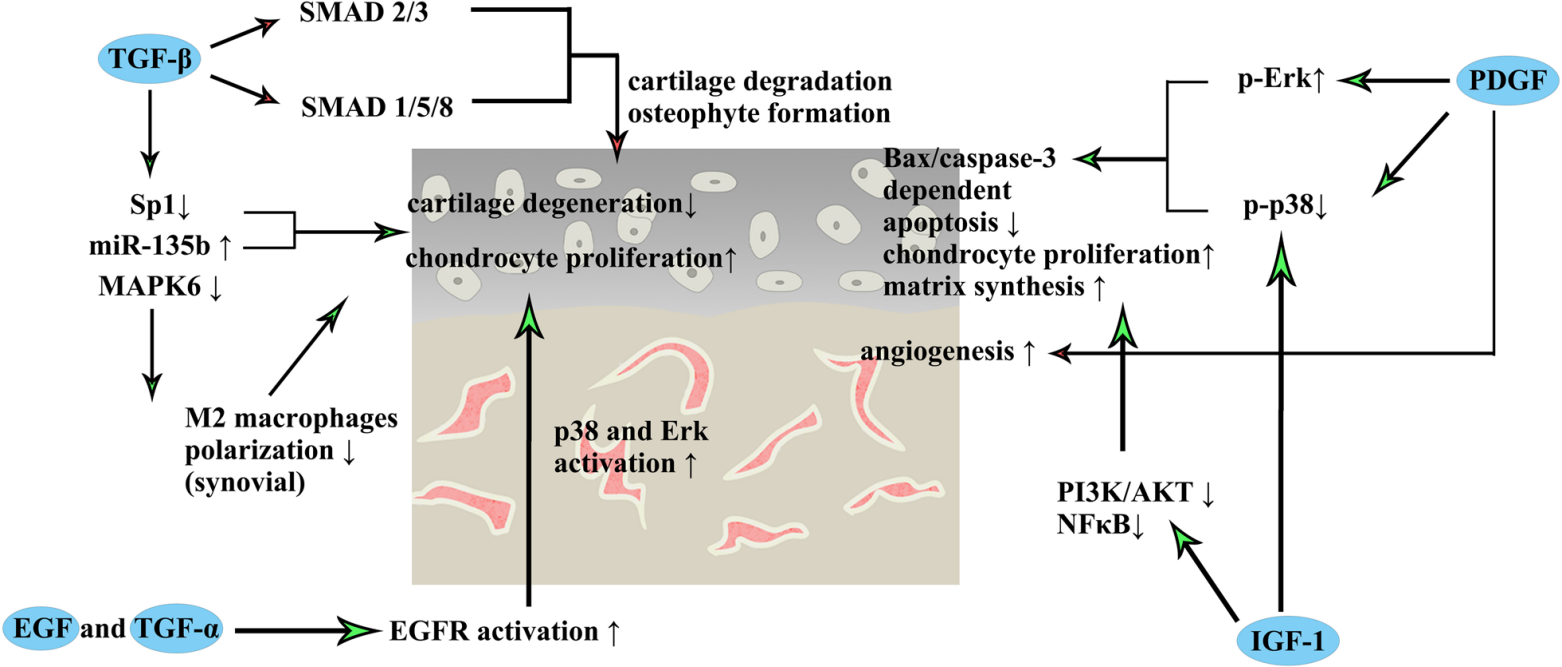
Mechanisms of cytokines in PRP that have positive effects on OA treatment

### TGF-β

TGF-β plays an important role in tissue repair and regeneration [23]. The TGF-β superfamily includes over 30 members, which can be divided into two ligand subfamilies [24]. These subfamilies, which are defined by sequence similarity and the activation of SMAD proteins, include the TGF-β/activin/nodal subfamily and the bone morphogenetic protein/growth and differentiation factor/Muellerian inhibiting substance subfamily [24]. TGF-β binds to TGF-β receptor type II and exerts its effects by bringing together pairs of type I and type II receptors on the cell surface [24]. TGF-β receptors are serine/threonine kinases, but type II receptors can induce type I receptor phosphorylation. TGF-β was significantly upregulated in osteophytes and cartilage in mice [25]. Moreover, TGF-β expression was increased in the joint synovium and subchondral bone in OA mouse models [26, 27]. Inhibiting endogenous TGF-β could decrease osteophyte formation and cartilage degradation in a mouse OA model [25, 27]. A previous review suggested that TGF-β could increase cartilage degradation and osteophyte formation and decrease cartilage maintenance through the TGF-β/SMAD2/3 and BMP/SMAD1/5/8 pathways, resulting in OA development [28]. However, these studies focused on endogenous TGF-β. Recent studies have gradually focused on the effects of exogenous TGF-β on OA, which is more suitable for elucidating the effect of TGF-β in PRP. Wang et al. treated OA with TGF-β1 (10 ng/mL)-induced mesenchymal stem cell-derived exosomes and found that it could decrease cartilage degeneration and enhance chondrocyte proliferation in rats [29]. Moreover, the researchers proved that the protective effects resulted from TGF-β1-mediated Sp1 downregulation and miR-135b upregulation [29]. Another study by these authors was published in 2021 and reported that TGF-β1 (10 ng/mL) was useful in the treatment of borrow mesenchymal stem cell exosomes, as it upregulated mir-135b, which suppressed the expression of MAPK6, resulting in M2 macrophage polarization in synovial tissue and reducing cartilage damage in OA rat models [30]. The results of Wang et al. seem to contradict to those of past studies. However, there are obvious differences in the methods that make a comparison unsuitable. The TGF-β1 concentration in PRP is approximately 200 ng/mL [31], which is much higher than that of endogenous TGF-β1 in the cartilage of OA patients. To the best of our knowledge, there is no direct evidence illustrating the relationship between endogenous and exogenous TGF-β and the effect of different concentrations of TGF-β in cartilage. However, these results showed that TGF-β had a positive effect on cartilage protection and that a high concentration of exogenous TGF-β released by mesenchymal stem cells could attenuate OA cartilage injury. Thus, the functions of TGF-β in PRP treatment of OA deserve to be further explored.

### PDGF

PDGF was first identified in platelets and includes four monomers: PDGF-A, -B, -C, and -D [32]. These four monomers constitute five types of dimers: PDGF-AA, -AB, -BB, -CC, and -DD [32]. PDGF exerts its effects by binding to PDGFR. PDGF-AA, -AB, -BB, and -CC can bind to PDGFRα, while PDGF-BB and -DD can bind to PDGFRβ [32]. Moreover, PDGF-AB, -BB, and -CC can stimulate the heterodimeric PDGFRα/β complex [32]. The PDGF family plays an important role in wound healing through its positive effects on mitosis, chemotaxis, and angiogenesis [33]. The main types of PDGF in PRP are PDGF-AA, -AB, and -BB [14]. Regarding its effects on chondrocytes, PDGF-AA has been reported to increase proteoglycan production in chondrocytes and promote cartilage repair in rabbits [34]. Moreover, a study published in 2019 showed that suppressing the production of PDGF-AA in subchondral bone through strenuous running downregulated the PDGF/AKT signaling pathway and promoted cartilage degeneration in mice [35]. However, there have been relatively fewer studies on PDGF-AA and -AB than on PDGF-BB. Many studies have reported that PDGF-BB exerts a protective effect on OA by promoting cartilage repair, decreasing inflammation, and inhibiting cartilage hypertrophy and osteophyte formation [21, 22, 36–38]. Our study was published in 2021 and showed that PDGF-BB could suppress chondrocyte apoptosis by upregulating Erk phosphorylation and inhibiting the p38/Bax/caspase-3 pathway in rats [21]. In addition, we discovered that PDGF-BB binding to PDGFRβ could enhance chondrocyte proliferation and cartilage matrix synthesis in rat OA models [22]. However, a study reported that the serum levels PDGF-BB increased in OA patients during the early stage [39]. Sun et al. showed that an elevated serum level of endogenous PDGF-BB contributed to OA development by stimulating angiogenesis and that knocking out pdgfb led to less damage in articular cartilage after medial meniscus destabilization surgery in mice [40]. These results can be explained as follows: (1) the compensatory increase in PDGF-BB is an early event in OA; (2) the serum concentration of PDGF-BB is approximately 20 ng/mL in mice, which may be much less than that in cartilage [39, 40]; and (3) gene knockout affects the overall expression of PDGF, which may involve more confounding factors and thus influence the objectivity of the results. Although the PDGF-BB concentration in PRP is approximately 10 ng/mL in human [31], a relatively high local concentration of endogenous PDGF can exert different effects. Therefore, PDGF-BB still presents high potential in protecting cartilage and preventing OA progression. However, whether PDGF-AA and PDGF-AB exert similar effects is unclear and warrants further research.

### EGF and TGF-α

The EGF family contains 11 structurally related proteins, including EGF, TGF-α, amphiregulin, epigen, heparin-binding EGF-like growth factor (HBEGF), epiregulin, betacellulin, and neuregulin [41]. These subfamilies have similar EGF-like motifs and exert effects via the Erb subclass of the receptor tyrosine kinase superfamily (EGFR) [42]. Previous studies have reported that EGFR activation can lead to cell proliferation, cell survival, and stem cell maintenance via the PI3K/AKT, MAPK, and JAK/STAT signaling pathways [43, 44]. The effects of EGF/TGF-α-EGFR activation have not been completely revealed. Long et al. reported that HBEGF expression was increased in a mouse OA model and human OA articular cartilage [45]. Moreover, 10–100 ng/mL HBEGF increased catabolic activity in normal chondrocytes and activation of the Erk and p38 signaling pathways in mice [45]. However, recent studies present different conclusions. Jia et al. showed that Egfr-deficient mice developed more severe OA than wild-type mice after surgical induction, as indicated by fewer superficial chondrocytes, less secretion of boundary lubrication, and weaker mechanical strength at the cartilage surface [46]. Moreover, previous studies suggested that activation of the EGFR signaling pathway under controlled conditions may promote the anabolic activity of articular cartilage and act as a viable strategy for articular cartilage repair and OA treatment in mice [47, 48]. A study conducted in 2021 on HBEGF-overexpressing mice investigated the effects of increasing EGFR activation during OA [49]. The researchers suggested that TGF-α and HBEGF were increased, which was associated with the formation of cell clusters after cartilage damage, but their endogenous expression was not sufficiently high for cartilage regeneration [49]. Therefore, whether increasing EGFR activity via TGF-α or HBEGF exert a protective effect on cartilage during OA needs further investigation.

### IGF-1

IGF-1 is a cytokine that regulates skeletal growth and development [50]. Most IGF-1 in circulation is produced by the liver in response to stimulation with growth hormone (GH) [50]. Moreover, it has been proven that chondrocytes produce IGF-1 [50, 51]. A previous study demonstrated that IGF could upregulate the synthesis of proteoglycan and collagen II and downregulate the synthesis of MMP-13 via the phosphorylation of ERK1/2 and AKT in rat endplate chondrocytes [52]. A recent study published in 2021 demonstrated that IGF-1 downregulated the p38, PI3K/AKT, and NF-κB signaling pathways to decrease reactive oxygen species production, resulting in antiapoptotic effects in OA rabbit models [53]. However, studies focused on the whole body concluded that serum IGF-1 levels were positively related to morbidity due to OA in humans [54–56]. On the other hand, some studies showed that OA patients exhibited increased serum GH levels and decreased serum IGF-1 levels [57, 58]. Serum IGF-1 mainly comes from the liver and is regulated by the GH/IGF-1 axis [59]. As a result, serum levels of IGF-1 and its regulation are a complex process and are difficult to examine. However, to evaluate the local treatment efficacy of IGF-1 or PRP, it is not required to know whether IGF-1 expression decreases in OA cartilage and whether IGF-1 exerts positive effects when directly administered to chondrocytes.

## Cytokines that have negative effects on OA treatment (Fig. 2)

**Fig. 2:**
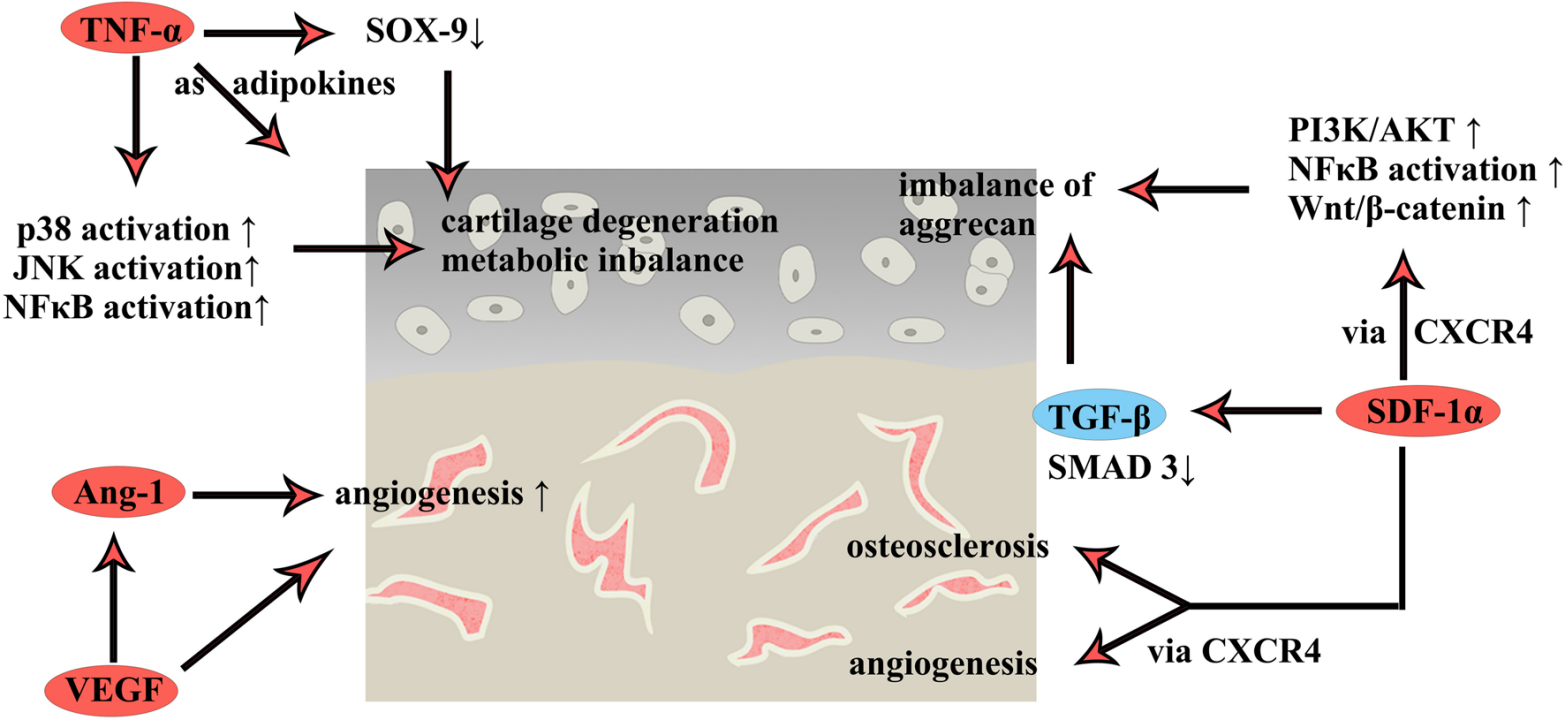
Mechanisms of cytokines in PRP that have negative effects on OA treatment

### VEGF

VEGF is the key survival factor for growth plate chondrocytes during embryonic development [60, 61] and plays an important role in endochondral ossification and skeletal development [62]. The VEGF family contains VEGF-A, -B, -C, -D, and placental growth factor. VEGF exerts its effects by binding to VEGF receptors (VEGFR)-1 (Flt-1), -2 (KDR), and -3 (Flt-4), which are tyrosine kinases that mediate subsequent signal transduction [63]. VEGFR-1 can bind with VEGF-A, -B, and placental growth factors; VEGFR-2 can bind with VEGF-A, -C, and -D; and VEGFR-3 can bind with VEGF-C and -D [64, 65]. During the progression of OA, the expression of VEGF increases in articular cartilage, the synovium, synovial fluid, subchondral bone, and serum, indicating that VEGF is a biomarker of OA, as indicated in the DisGeNET database [63]. The main effects of VEGF during OA progression are believed to be induced through angiogenesis. Previous studies have reported that VEGF promotes vascular invasion in not only cartilage [66–68] but also the synovium and meniscus [68–72]. As summarized in a published review, VEGF binding to VEGFR led to cartilage degeneration, osteophyte formation, subchondral bone cysts and sclerosis, synovitis, and pain [63]. Inhibiting the effects of VEGF and VEGFR in OA animal models could attenuate the progression of OA in rabbits [73]. Based on these studies, VEGF may exert negative effects on PRP treatment of OA due to the promotion of angiogenesis and cartilage degeneration. A study published in 2022 showed that VEGF-attenuated PRP-mediated improvements in OA in rats by preventing unwanted biological activity using growth factor-binding microspheres [74]. These studies suggested that VEGF-mediated attenuation of PRP exhibited better tissue repair effects than traditional PRP treatment [74]. This study suggests that if a synthetic cytokine mixture was developed to replace PRP, VEGF can be ignored.

### TNF-α

TNF-α is a pleiotropic cytokine produced by a variety of cells, such as adipocytes, lymphocytes, endothelial cells, osteoblasts, and smooth muscle cells, that plays important roles in homeostasis and disease pathogenesis [75, 76]. TNF-α exerts its effects by binding to TNF receptors 1 and 2, and it has well-known antitumor functions [77]. In addition, it also plays a role in stimulating lymphocytes, monocytes, and neutrophils, modulating temperature, and combating infection [78–80]. Regarding its role in OA, TNF-α can increase the production of inflammatory cytokines, MMPs, and prostaglandin 9 and inhibit the synthesis of proteoglycan and collagen II in chondrocytes, leading to the degeneration of cartilage and promoting OA progression in rats [81, 82]. Moreover, TNF-α can decrease the expression of the transcription factor SOX-9 and reduce the efficiency of the respiratory chain in mice and normal human chondrocytes [83, 84]. Mechanistically, TNF-α can activate the NF-κB, JNK, and p38 signaling pathways in chondrocytes to promote the occurrence of OA in humans and rats [83, 85–87]. Recently, researchers have focused on the adipokine role of TNF-α, which has been proven to regulate the metabolic balance in joints by influencing the expression of cytokines, chemokines, matrix-degrading enzymes, and cell growth and differentiation factors in rats [88]. Moreover, an increase in serum levels of TNF-α was discovered in obese adults [89]. It has been discovered that obesity is positively related to the occurrence of musculoskeletal disorders, and adipocytokines secreted by adipose tissues can promote the occurrence of OA [90, 91]. The activation of the adipocytokine signaling pathway by TNF-α may explain the close relationship between obesity and OA [77, 92]. Considering its proinflammatory effect and the high probability of negative effects on OA, the levels of TNF-α in PRP should be monitored when PRP is used to treat OA.

### Ang-1

Ang-1 is a cytokine that mainly regulates angiogenesis [93, 94]. The effects of Ang-1 are triggered by VEGF, followed by binding to its receptors Tie1 and Tie2 [93, 94]. When VEGF-mediated formation of immature vessels occurs, Ang-1 stabilizes the new blood vessels by recruiting neighboring mesenchymal cells and promoting mesenchymal cell differentiation into vascular smooth muscle cells in mice [93]. The level of Ang-1 is increased in the OA joint synovium, but there are no significant differences in OA osteoblasts in normal joints [95, 96]. The level of Ang-1 in OA chondrocytes has not yet been discovered. Although there is little evidence on the effects of Ang-1 on OA, previous studies have proven that it is a downstream factor of VEGF, indicating that its status in PRP treatment of OA may be similar to that of VEGF.

### SDF-1α

SDF-1, which is also known as CXC motif chemokine ligand 12 (CXCL12), is a member of the CXC motif family [97]. It exerts effects by binding to its receptor CXC chemokine receptor types 4 and 7 [98]. SDF-1α is a subtype of SDF-1. SDF-1 is involved in the regulation of cell differentiation, cell distribution, cell proliferation, cell adhesion, gene expression, neovascularization in multiple cells and tissues, and embryonic development [99]. SDF-1α promotes chondrocyte proliferation via Erk- and NF-κB-mediated cyclin D1 upregulation in primary mouse chondrocytes [100]. Moreover, some studies suggest that the downregulation of SDF-1 could inhibit proliferation in human chondrocyte lines [101, 102]. The potential mechanisms of SDF-1 in OA cartilage are complicated. SDF-1 can be produced by the OA joint synovium and bind to CXCR4 and CXCR7, which are expressed on chondrocytes [103–105]. The SDF-1/CXCR4 axis can activate the PI3K/Akt, NF-κB, and Wnt/β-catenin pathways and suppress the TGF-β/Smad3 pathway, leading to an imbalance in aggrecan in rat OA cartilage [106, 107]. Compared to that in normal joints, the level of SDF-1 in subchondral bone is increased in OA, which results in the formation of subchondral osteosclerosis in humans [108, 109]. A study published in 2021 noted that inhibiting the SDF-1/CXCR4 axis could alleviate abnormal bone formation and angiogenesis, resulting in improvements in the subchondral bone microenvironment in OA mouse models [110]. Exogenous SDF-1α loaded on nanofibrous hyaluronic acid scaffolds has been applied to minipig joints [111]. This study reported that the articular cartilage repair capacity of SDF-1α was limited and could weaken the cartilage repair effects of TGF-β [111]. Although some studies have reported that SDF-1α could promote chondrocyte proliferation, its negative effects on the extracellular matrix and subchondral bone limit its potential use in the treatment of OA.

## Cytokines with unknown effects on OA

### CTGF

CTGF is composed of five domains: the secretory signal peptide, IGF binding protein (IGFBP), von Willebrand factor type C repeat (VWC), thrombospondin type 1 repeat (TSP1), and C-terminal cystine-knot (CT) modules [112]. IGFBP binds to IGF and enhances its affinity for IGFR [113]. The TSP1 domain binds to various molecules, including collagen V, fibronectin, TGF-β, and VEGF. Previous studies have reported that the TSP1 domain could upregulate the TGF-β signaling pathway and downregulate the VEGF pathway [114, 115]. Regarding its effects on OA, CTGF was overexpressed in OA cartilage and synovial fluid in mice and humans [116, 117]. CTGF could increase the production of inflammatory factors such as MMP3 and promote cartilage degeneration by activating NF-κB in mouse OA chondrocytes [117]. As mentioned earlier, PRP contains multiple biological factors, including CTGF, IGF, TGF-β, and VEGF. Whether exogenous CTGF in PRP exerts positive or negative effects on OA in the presence of IGF, TGF-β, or VEGF remains unclear. However, studies have shown that CTGF promotes the effects of TGF-β and inhibits the effects of VEGF, indicating that exogenous administration could benefit OA. These findings suggest that CTGF exerts complex effects due to its multiple biological targets in different contexts. Regarding the effect of PRP on OA, further studies need to be conducted to confirm this hypothesis.

### FGF

FGF is a mitogen that regulates cellular migration, proliferation, differentiation, and survival. FGF exerts its effects by binding to the FGF receptor (FGFR), triggering conformational changes in FGFR and leading to the phosphorylation of tyrosine residues on the cytoplasmic side of FGFR [118]. The FGF/FGFR signaling pathway exerts important effects on the development and homeostasis of articular cartilage. One study revealed that the expression of FGFR1 was upregulated, and the expression of FGFR3 was downregulated in human OA cartilage [119]. The FGFR1 signaling pathway could accelerate matrix degradation by upregulating the transcription factors RUNX-2 and ELK-1 [120]. Previous studies showed that inhibiting FGR1 expression could decrease the synthesis of MMP13 and reduce the progression of cartilage degeneration [121]. Regarding the FGFR3 signaling pathway, previous studies have proven that it could promote anabolism in chondrocytes, decrease the production of inflammatory factors, and decrease hypertrophic differentiation by downregulating the IHH signaling pathway [122–124]. The results that FGFR1 and FGFR3 in OA were antipodals seemed contradictory because both factors were activated by FGF [125]. Moreover, FGFs are a large family associated with multiple reactions and effects, constituting a complex network [125, 126]. As a result, whether FGF exerts any effects on PRP treatment of OA remains unclear. Whether changing the mode of action of FGF by triggering FGFR3 can lead to a positive effect of PRP needs to be explored further.

### HGF

HGF is a cytokine that is secreted by multiple cells, such as platelets, mesenchymal cells, and endothelial cells. It was first identified as a promoter of liver regeneration [127, 128]. HGF an exert its effects by being converted to an active heterodimeric form by HGF activator (HGFA) and then binding to its receptor c-Met, which is a transmembrane tyrosine kinase [129]. Previous studies have reported that HGF could promote the organization and reconstruction of tissues, indicating its potential for tissue repair [130]. Moreover, HGF levels were upregulated in chondrocytes and the plasma of OA patients [127, 131]. HGF has also been proven to modulate extracellular matrix components and increase the level of TGF-β in Leydig cells [132]. A study published in 2022 reported that HGF overexpression could activate the HGF/c-Met signaling pathway and induce the degradation of the extracellular cellular matrix, promoting the progression of OA in mice [133]. However, research focusing on the association between HGF and OA is limited, and the exact relationship between OA and HGF and exogenous HGF and whether it benefits OA patients remain unclear.

## Conclusion

**Fig. 3:**
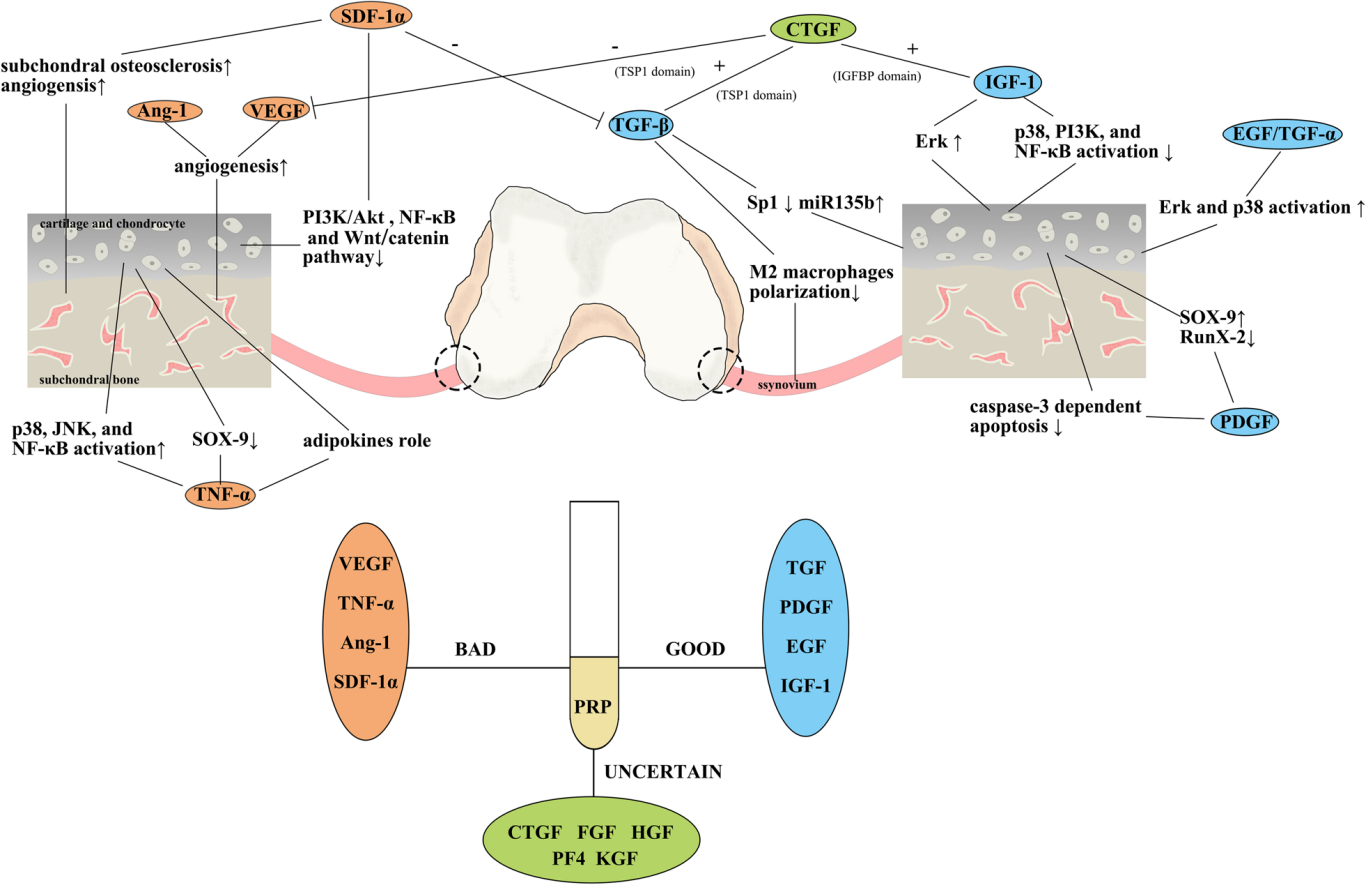
The effects of cytokines contained in PRP on OA. Cytokines can exert their effects by reacting with chondrocytes/cartilage, subchondral bone, and the synovium. In summary, TGF, PDGF, EGF and IGF-1 may exert positive effects on OA treatment; VEGF, TNF-α, and SDF-1α may exert negative effects on OA treatment; and the effects of CTGF, FGF, HGF, PF4 and KGF on OA are still unknown

This is the first review to summarize the effects of cytokines in PRP on OA. However, because there are few available studies on PF4 and KGF, they have not been included in this review. The cytokines that are potentially beneficial or detrimental for OA treatment are summarized in Table 1. As described previously, PRP components are complex and could can various effects via complex mechanisms. The available evidence suggests that TGF-α and β, PDGF, EGF, and IGF-1 have potential for treating OA, while VEGF, TNF-α, Ang-1, and SDF-1α can induce negative effects on OA patients (Fig. 3). In addition, the effects of CTGF, FGF, HGF, PF4, and KGF on OA remain unclear (Fig. 3). Based on these studies, we concluded that not all cytokines in PRP can benefit OA patients and that the therapeutic effects of PRP have significant potential for improvement. Moreover, with consideration to the complicated preparation of PRP and the restrictions requiring repeated operations, a cytokine mixture containing these beneficial cytokines at appropriate concentrations and eliminating detrimental cytokines can be a strategy to replace PRP. This article mainly focused on OA. The same cytokines may exert different effects on other musculoskeletal disorders. For example, angiogenesis is generally considered a key process in OA and inhibits OA recovery, which explains why VEGF and Ang-1 are not beneficial for OA. However, angiogenesis is important for healing of rotator cuff tears [134, 135], indicating that VEGF and Ang-1 are beneficial cytokines for rotator cuff tears. Thus, the proposed replacement scheme can provide an opportunity to adjust the components for different diseases to induce the best benefits.

**Table 1:** Summary of the cytokines that are beneficial or detrimental for OA treatment

Cytokines	Years	Effect	Model	Species	Description
TGF-β	2002 [21]	Beneficial	OA	Mouse	Inhibition of endogenous TGF-β decreased osteophyte formation and cartilage degradation
	2013 [23]		OA	Mouse	
	2018 [25]	Beneficial	OA	Rat	TGF-β decreased cartilage degeneration and enhanced chondrocyte proliferation
	2021 [26]		OA	Rat	TGF-β suppressed MAPK6 expression and led to M2 macrophages polarization in the synovium
PDGF-AA	2019 [31]	Might be detrimental	Cartilage degeneration	Mice	PDGF-AA in subchondral bone led to articular cartilage degeneration
	1991 [30]	Might be beneficial	cartilage lesion	Rabbit	PDGF-AA could increase proteoglycan production in chondrocytes and promote cartilage repair
PDGF-BB	2020 [36]	Detrimental	OA	Mice	Elevated serum levels of endogenous PDGF-BB contributed to OA development
	2021 [17]	Beneficial	OA	Rat	PDGF-BB could inhibit p38/Bax/caspase-3-dependent chondrocyte apoptosis
	2022 [18]		OA	Rat	PDGF-BB could enhance chondrocyte proliferation and cartilage matrix synthesis
EGF and TGF-α	2015 [41]	Might be detrimental	Normal chondrocyte	Mouse	HBEGF-treated normal chondrocyte showed increased catabolic activity
	2016 [42]	Beneficial	OA	Mouse	Egfr-deficient mice developed more severe OA than wild-type mice
	2014 [43]		OA	Mouse	Activation of the EGFR signaling pathway promoted the anabolic activity of articular cartilage
	2013 [44]		OA	Mouse	
	2021 [45]		OA	Mouse	TGF-α and HBEGF were increased during the formation of cell clusters after cartilage damage
IGF-1	2009 [48]	Might be beneficial	Normal chondrocyte	Rat	IGF could upregulate the synthesis of proteoglycan and collagen II and downregulate the synthesis of MMP-13 in rat endplate chondrocytes
	2021 [49]	beneficial	OA	Rabbit	IGF-1 decreased reactive oxygen species production, resulting in antiapoptotic effects
VEGF	2014 [69]	Beneficial	OA	Rabbit	Inhibition of the effects of VEGF and VEGFR in OA animal models could attenuate the progression of OA
	2022 [70]		OA	Rat	VEGF-depleted PRP improved the healing of OA
TNF-α	2005 [77]	Detrimental	OA	rat	TNF-α could increase the production of inflammatory cytokines and inhibit the synthesis of proteoglycan and collagen II in chondrocytes, leading to the degeneration of cartilage
	2004 [78]		OA	Rat	
	2000 [79]	Might be detrimental	Normal chondrocyte	Rat	TNF-α could decrease the expression of the transcription factor SOX-9 and reduce the efficiency of the respiratory chain
	2006 [80]		Normal chondrocyte	Human	TNF-α could decrease the expression of the transcription factor SOX-9 and reduce the efficiency of the respiratory chain
Ang-1	1999 [89]	Might be detrimental	Normal mesenchymal cells	Mouse	Ang-1 stabilized new blood vessels by recruiting neighboring mesenchymal cells during VEGF-mediated vessels formation
SDF-1α	2019 [102]	Detrimental	OA	Rat	The SDF-1/CXCR4 axis could lead to an imbalance in aggrecan in rat OA cartilage
	2016 [103]		OA	Rat	
	2006 [104]		OA	Human	SDF-1 in subchondral bone was increased in OA, which resulted in the formation of subchondral osteosclerosis
	2017 [105]		OA	Human	
	2021 [106]		OA	Mouse	SDF-1/CXCR4 axis could alleviate abnormal bone formation and angiogenesis

The evaluative criteria of the “Effect” item are shown below

Detrimental: Cytokines have negative effects on OA treatment

Might be detrimental: Cytokines could harm the chondrocytes or other cells or tissues related to OA

Might be beneficial: Cytokines could benefit chondrocytes or other cells or tissues related to OA

Beneficial: Cytokines have positive effects on OA treatment

## Data Availability

The datasets used and/or analyzed during the current study are available from the corresponding author on reasonable request.

## Abbreviations

OA: Osteoarthritis
PRP: Platelet-rich plasma
PDGF: Platelet-derived growth factors
TGF: Transforming growth factor
VEGF: Vascular endothelial growth factor
EGF: Epidermal growth factor
FGF: Fibroblast growth factor
CTGF: Connective tissue growth factor
IGF: Insulin-like growth factor
HGF: Hepatocyte growth factor
KGF: Keratinocyte growth factor
Ang-1: Angiopoietin-1
PF: Platelet factor
SDF: Stromal cell derived factor
TNF: Tumor necrosis factor

## References

[1] Hunter DJ, Bierma-Zeinstra S (2019). Osteoarthritis. Lancet.

[2] Martel-Pelletier J, Barr AJ, Cicuttini FM, Conaghan PG, Cooper C, Goldring MB, Goldring SR, Jones G, Teichtahl AJ, Pelletier JP (2016). Osteoarthritis. Nat Rev Dis Primers.

[3] Zhang W, Nuki G, Moskowitz RW, Abramson S, Altman RD, Arden NK, Bierma-Zeinstra S, Brandt KD, Croft P, Doherty M (2010). OARSI recommendations for the management of hip and knee osteoarthritis: part III: changes in evidence following systematic cumulative update of research published through January 2009. Osteoarthr Cartil.

[4] Bruyère O, Cooper C, Pelletier JP, Branco J, Luisa Brandi M, Guillemin F, Hochberg MC, Kanis JA, Kvien TK, Martel-Pelletier J (2014). An algorithm recommendation for the management of knee osteoarthritis in Europe and internationally: a report from a task force of the European society for clinical and economic aspects of osteoporosis and osteoarthritis (ESCEO). Semin Arthritis Rheum.

[5] Hochberg MC, Altman RD, April KT, Benkhalti M, Guyatt G, McGowan J, Towheed T, Welch V, Wells G, Tugwell P (2012). American college of rheumatology 2012 recommendations for the use of nonpharmacologic and pharmacologic therapies in osteoarthritis of the hand, hip, and knee. Arthritis Care Res.

[6] Hermann W, Lambova S, Muller-Ladner U (2018). Current treatment options for osteoarthritis. Curr Rheumatol Rev.

[7] Fuggle NR, Cooper C, Oreffo ROC, Price AJ, Kaux JF, Maheu E, Cutolo M, Honvo G, Conaghan PG, Berenbaum F (2020). Alternative and complementary therapies in osteoarthritis and cartilage repair. Aging Clin Exp Res.

[8] Gupta A, Jeyaraman M, Maffulli N (2022). Common medications which should be stopped prior to platelet-rich plasma injection. Biomedicines.

[9] Andia I, Maffulli N (2019). Blood-derived products for tissue repair/regeneration. Int J Mol Sci.

[10] Andia I, Maffulli N (2018). Some patients (and some of us) respond better to some biological therapies: the as yet unsolved conundrum. J Orthop Traumatol: Off J Italian Soc Orthop Traumatol.

[11] Weibrich G, Hansen T, Kleis W, Buch R, Hitzler WE (2004). Effect of platelet concentration in platelet-rich plasma on peri-implant bone regeneration. Bone.

[12] Graziani F, Ivanovski S, Cei S, Ducci F, Tonetti M, Gabriele M (2006). The in vitro effect of different PRP concentrations on osteoblasts and fibroblasts. Clin Oral Implant Res.

[13] Anitua E, Sánchez M, Nurden AT, Nurden P, Orive G, Andía I (2006). New insights into and novel applications for platelet-rich fibrin therapies. Trends Biotechnol.

[14] Everts P, Onishi K, Jayaram P, Lana JF, Mautner K (2020). Platelet-rich plasma: new performance understandings and therapeutic considerations in 2020. Int J Mol Sci.

[15] Filardo G, Di Matteo B, Kon E, Merli G, Marcacci M (2018). Platelet-rich plasma in tendon-related disorders: results and indications. Knee Surg Sports Traumatol Arthroscop: Off J ESSKA.

[16] Xuan Z, Yu W, Dou Y, Wang T (2020). Efficacy of platelet-rich plasma for low back pain: a systematic review and meta-analysis. J Neurol Surg Part A, Cent Eur Neurosurg.

[17] Andia I, Maffulli N (2018). A contemporary view of platelet-rich plasma therapies: moving toward refined clinical protocols and precise indications. Regen Med.

[18] Belk JW, Kraeutler MJ, Houck DA, Goodrich JA, Dragoo JL, McCarty EC (2021). Platelet-rich plasma versus hyaluronic acid for knee osteoarthritis: a systematic review and meta-analysis of randomized controlled trials. Am J Sports Med.

[19] Zhao J, Huang H, Liang G, Zeng LF, Yang W, Liu J (2020). Effects and safety of the combination of platelet-rich plasma (PRP) and hyaluronic acid (HA) in the treatment of knee osteoarthritis: a systematic review and meta-analysis. BMC Musculoskelet Disord.

[20] Migliorini F, Driessen A, Quack V, Sippel N, Cooper B, Mansy YE, Tingart M, Eschweiler J (2021). Comparison between intra-articular infiltrations of placebo, steroids, hyaluronic and PRP for knee osteoarthritis: a Bayesian network meta-analysis. Arch Orthop Trauma Surg.

[21] Zhu P, Wang Z, Sun Z, Liao B, Cai Y (2021). Recombinant platelet-derived growth factor-BB alleviates osteoarthritis in a rat model by decreasing chondrocyte apoptosis in vitro and in vivo. J Cell Mol Med.

[22] Cai Y, Wang Z, Liao B, Sun Z, Zhu P (2023). Anti-inflammatory and chondroprotective effects of platelet-derived growth factor-BB on osteoarthritis rat models. J Gerontol: Ser A.

[23] Hinck AP (2012). Structural studies of the TGF-βs and their receptors - insights into evolution of the TGF-β superfamily. FEBS Lett.

[24] Shi Y, Massagué J (2003). Mechanisms of TGF-beta signaling from cell membrane to the nucleus. Cell.

[25] Scharstuhl A, Glansbeek HL, van Beuningen HM, Vitters EL, van der Kraan PM, van den Berg WB (2002). Inhibition of endogenous TGF-beta during experimental osteoarthritis prevents osteophyte formation and impairs cartilage repair. J Immunol.

[26] Bakker AC, van de Loo FA, van Beuningen HM, Sime P, van Lent PL, van der Kraan PM, Richards CD, van den Berg WB (2001). Overexpression of active TGF-beta-1 in the murine knee joint: evidence for synovial-layer-dependent chondro-osteophyte formation. Osteoarth Cartil.

[27] Zhen G, Wen C, Jia X, Li Y, Crane JL, Mears SC, Askin FB, Frassica FJ, Chang W, Yao J (2013). Inhibition of TGF-β signaling in mesenchymal stem cells of subchondral bone attenuates osteoarthritis. Nat Med.

[28] Zhai G, Doré J, Rahman P (2015). TGF-β signal transduction pathways and osteoarthritis. Rheumatol Int.

[29] Wang R, Xu B, Xu H (2018). TGF-β1 promoted chondrocyte proliferation by regulating Sp1 through MSC-exosomes derived miR-135b. Cell Cycle.

[30] Wang R, Xu B (2021). TGF-β1-modified MSC-derived exosomal miR-135b attenuates cartilage injury via promoting M2 synovial macrophage polarization by targeting MAPK6. Cell Tissue Res.

[31] Masuki H, Okudera T, Watanebe T, Suzuki M, Nishiyama K, Okudera H, Nakata K, Uematsu K, Su CY, Kawase T (2016). Growth factor and pro-inflammatory cytokine contents in platelet-rich plasma (PRP), plasma rich in growth factors (PRGF), advanced platelet-rich fibrin (A-PRF), and concentrated growth factors (CGF). Int J Implant Dent.

[32] Fredriksson L, Li H, Eriksson U (2004). The PDGF family: four gene products form five dimeric isoforms. Cytokine Growth Factor Rev.

[33] Alvarez RH, Kantarjian HM, Cortes JE (2006). Biology of platelet-derived growth factor and its involvement in disease. Mayo Clin Proc.

[34] Peracchia F, Ferrari G, Poggi A, Rotilio D (1991). IL-1 beta-induced expression of PDGF-AA isoform in rabbit articular chondrocytes is modulated by TGF-beta 1. Exp Cell Res.

[35] Yao Z, Chen P, Wang S, Deng G, Hu Y, Lin Q, Zhang X, Yu B (2019). Reduced PDGF-AA in subchondral bone leads to articular cartilage degeneration after strenuous running. J Cell Physiol.

[36] Razmara M, Eger G, Rorsman C, Heldin CH, Lennartsson J (2012). MKP3 negatively modulates PDGF-induced Akt and Erk5 phosphorylation as well as chemotaxis. Cell Signal.

[37] Xiong LL, Xue LL, Jiang Y, Ma Z, Jin Y, Wang YC, Wang YY, Xia QJ, Zhang Y, Hu Q (2019). Suppression of PDGF induces neuronal apoptosis after neonatal cerebral hypoxia and ischemia by inhibiting P-PI3K and P-AKT signaling pathways. Brain Res.

[38] Setiawan I, Suyasa IK, Astawa P, Dusak IWS, Kawiyana IKS, Aryana I (2019). Recombinant platelet derived growth factor-BB and hyaluronic acid effect in rat osteoarthritis models. J Orthop.

[39] Kisand K, Tamm AE, Lintrop M, Tamm AO (2018). New insights into the natural course of knee osteoarthritis: early regulation of cytokines and growth factors, with emphasis on sex-dependent angiogenesis and tissue remodeling. A Pilot Study Osteoarth Cartil.

[40] Su W, Liu G, Liu X, Zhou Y, Sun Q, Zhen G, Wang X, Hu Y, Gao P, Demehri S, Cao X. Angiogenesis stimulated by elevated PDGF-BB in subchondral bone contributes to osteoarthritis development. JCI Insight 2020;5(8):e135446. 10.1172/jci.insight.135446. 10.1172/jci.insight.135446 PMC7205438 32208385

[41] Abud HE, Chan WH, Jardé T (2021). Source and impact of the EGF family of ligands on intestinal stem cells. Front Cell Develop Biol.

[42] Rayego-Mateos S, Rodrigues-Diez R, Morgado-Pascual JL, Valentijn F, Valdivielso JM, Goldschmeding R, Ruiz-Ortega M (2018). Role of epidermal growth factor receptor (EGFR) and Its ligands in kidney inflammation and damage. Med Inflamm.

[43] Singh AB, Harris RC (2005). Autocrine, paracrine and juxtacrine signaling by EGFR ligands. Cell Signal.

[44] Iwakura Y, Nawa H (2013). ErbB1-4-dependent EGF/neuregulin signals and their cross talk in the central nervous system: pathological implications in schizophrenia and Parkinson's disease. Front Cell Neurosci.

[45] Long DL, Ulici V, Chubinskaya S, Loeser RF (2015). Heparin-binding epidermal growth factor-like growth factor (HB-EGF) is increased in osteoarthritis and regulates chondrocyte catabolic and anabolic activities. Osteoarth Cartil.

[46] Jia H, Ma X, Tong W, Doyran B, Sun Z, Wang L, Zhang X, Zhou Y, Badar F, Chandra A (2016). EGFR signaling is critical for maintaining the superficial layer of articular cartilage and preventing osteoarthritis initiation. Proc Natl Acad Sci USA.

[47] Pest MA, Russell BA, Zhang YW, Jeong JW, Beier F (2014). Disturbed cartilage and joint homeostasis resulting from a loss of mitogen-inducible gene 6 in a mouse model of joint dysfunction. Arthritis Rheumatol.

[48] Shepard JB, Jeong JW, Maihle NJ, O'Brien S, Dealy CN (2013). Transient anabolic effects accompany epidermal growth factor receptor signal activation in articular cartilage in vivo. Arthritis Res Ther.

[49] Wei Y, Luo L, Gui T, Yu F, Yan L, Yao L, Zhong L, Yu W, Han B, Patel JM (2021). Targeting cartilage EGFR pathway for osteoarthritis treatment. Sci Trans Med.

[50] Laron Z (2001). Insulin-like growth factor 1 (IGF-1): a growth hormone. Mol Pathol: MP.

[51] Nilsson A, Isgaard J, Lindahl A, Dahlström A, Skottner A, Isaksson OG (1986). Regulation by growth hormone of number of chondrocytes containing IGF-I in rat growth plate. Science.

[52] Zhang M, Zhou Q, Liang QQ, Li CG, Holz JD, Tang D, Sheu TJ, Li TF, Shi Q, Wang YJ (2009). IGF-1 regulation of type II collagen and MMP-13 expression in rat endplate chondrocytes via distinct signaling pathways. Osteoarth Cartil.

[53] Hossain MA, Adithan A, Alam MJ, Kopalli SR, Kim B, Kang CW, Hwang KC, Kim JH (2021). IGF-1 facilitates cartilage reconstruction by regulating PI3K/AKT, MAPK, and NF-kB signaling in rabbit osteoarthritis. J Inflamm Res.

[54] Claessen KM, Ramautar SR, Pereira AM, Smit JW, Biermasz NR, Kloppenburg M (2012). Relationship between insulin-like growth factor-1 and radiographic disease in patients with primary osteoarthritis: a systematic review. Osteoarth Cartil.

[55] Lloyd ME, Hart DJ, Nandra D, McAlindon TE, Wheeler M, Doyle DV, Spector TD (1996). Relation between insulin-like growth factor-I concentrations, osteoarthritis, bone density, and fractures in the general population: the Chingford study. Ann Rheum Dis.

[56] Hartley A, Sanderson E, Paternoster L, Teumer A, Kaplan RC, Tobias JH, Gregson CL (2021). Mendelian randomization provides evidence for a causal effect of higher serum IGF-1 concentration on risk of hip and knee osteoarthritis. Rheumatology.

[57] Denko CW, Boja B, Moskowitz RW (1990). Growth promoting peptides in osteoarthritis: insulin, insulin-like growth factor-1, growth hormone. J Rheumatol.

[58] Denko CW, Boja B, Moskowitz RW (1996). Growth factors, insulin-like growth factor-1 and growth hormone, in synovial fluid and serum of patients with rheumatic disorders. Osteoarth Cartil.

[59] Dixit M, Poudel SB, Yakar S (2021). Effects of GH/IGF axis on bone and cartilage. Mol Cell Endocrinol.

[60] Zelzer E, Mamluk R, Ferrara N, Johnson RS, Schipani E, Olsen BR (2004). VEGFA is necessary for chondrocyte survival during bone development. Development.

[61] Maes C, Araldi E, Haigh K, Khatri R, Van Looveren R, Giaccia AJ, Haigh JJ, Carmeliet G, Schipani E (2012). VEGF-independent cell-autonomous functions of HIF-1α regulating oxygen consumption in fetal cartilage are critical for chondrocyte survival. J Bone Min Res: Off J Am Soc Bone Min Res.

[62] Duan X, Murata Y, Liu Y, Nicolae C, Olsen BR, Berendsen AD (2015). Vegfa regulates perichondrial vascularity and osteoblast differentiation in bone development. Development.

[63] Hamilton JL, Nagao M, Levine BR, Chen D, Olsen BR, Im HJ (2016). Targeting VEGF and Its receptors for the treatment of osteoarthritis and associated pain. J Bone Min Res: Off J Am Soc Bone Min Res.

[64] Ferrara N, Gerber HP, LeCouter J (2003). The biology of VEGF and its receptors. Nat Med.

[65] Olsson AK, Dimberg A, Kreuger J, Claesson-Welsh L (2006). VEGF receptor signalling - in control of vascular function. Nat Rev Mol Cell Biol.

[66] Walsh DA, McWilliams DF, Turley MJ, Dixon MR, Fransès RE, Mapp PI, Wilson D (2010). Angiogenesis and nerve growth factor at the osteochondral junction in rheumatoid arthritis and osteoarthritis. Rheumatology.

[67] Fransès RE, McWilliams DF, Mapp PI, Walsh DA (2010). Osteochondral angiogenesis and increased protease inhibitor expression in OA. Osteoarth Cartil.

[68] Walsh DA, Bonnet CS, Turner EL, Wilson D, Situ M, McWilliams DF (2007). Angiogenesis in the synovium and at the osteochondral junction in osteoarthritis. Osteoarth Cartil.

[69] Haywood L, McWilliams DF, Pearson CI, Gill SE, Ganesan A, Wilson D, Walsh DA (2003). Inflammation and angiogenesis in osteoarthritis. Arthritis Rheum.

[70] Giatromanolaki A, Sivridis E, Athanassou N, Zois E, Thorpe PE, Brekken RA, Gatter KC, Harris AL, Koukourakis IM, Koukourakis MI (2001). The angiogenic pathway "vascular endothelial growth factor/flk-1(KDR)-receptor" in rheumatoid arthritis and osteoarthritis. J Pathol.

[71] Im HJ, Kim JS, Li X, Kotwal N, Sumner DR, van Wijnen AJ, Davis FJ, Yan D, Levine B, Henry JL (2010). Alteration of sensory neurons and spinal response to an experimental osteoarthritis pain model. Arthritis Rheum.

[72] Ashraf S, Wibberley H, Mapp PI, Hill R, Wilson D, Walsh DA (2011). Increased vascular penetration and nerve growth in the meniscus: a potential source of pain in osteoarthritis. Ann Rheum Dis.

[73] Nagai T, Sato M, Kobayashi M, Yokoyama M, Tani Y, Mochida J (2014). Bevacizumab, an anti-vascular endothelial growth factor antibody, inhibits osteoarthritis. Arthritis Res Ther.

[74] Lee JS, Guo P, Klett K, Hall M, Sinha K, Ravuri S, Huard J, Murphy WL (2022). VEGF-attenuated platelet-rich plasma improves therapeutic effect on cartilage repair. Biomater Sci.

[75] Tracey KJ, Cerami A (1993). Tumor necrosis factor, other cytokines and disease. Annu Rev Cell Biol.

[76] Tracey KJ, Cerami A (1994). Tumor necrosis factor: a pleiotropic cytokine and therapeutic target. Annu Rev Med.

[77] Wang T, He C (2018). Pro-inflammatory cytokines: the link between obesity and osteoarthritis. Cytokine Growth Factor Rev.

[78] Clohisy JC, Teitelbaum S, Chen S, Erdmann JM, Abu-Amer Y (2002). Tumor necrosis factor-alpha mediates polymethylmethacrylate particle-induced NF-kappaB activation in osteoclast precursor cells. J Orthop Res: Off Publ Orthop Res Soc.

[79] Merkel KD, Erdmann JM, McHugh KP, Abu-Amer Y, Ross FP, Teitelbaum SL (1999). Tumor necrosis factor-alpha mediates orthopedic implant osteolysis. Am J Pathol.

[80] Jiranek WA, Machado M, Jasty M, Jevsevar D, Wolfe HJ, Goldring SR, Goldberg MJ, Harris WH (1993). Production of cytokines around loosened cemented acetabular components. Analysis with immunohistochemical techniques and in situ hybridization. J Bone Joint Surg Am.

[81] Klooster AR, Bernier SM (2005). Tumor necrosis factor alpha and epidermal growth factor act additively to inhibit matrix gene expression by chondrocyte. Arthritis Res Ther.

[82] Goldring SR, Goldring MB (2004). The role of cytokines in cartilage matrix degeneration in osteoarthritis. Clin Orthop Related Res.

[83] Murakami S, Lefebvre V, de Crombrugghe B (2000). Potent inhibition of the master chondrogenic factor Sox9 gene by interleukin-1 and tumor necrosis factor-alpha. J Biol Chem.

[84] López-Armada MJ, Caramés B, Martín MA, Cillero-Pastor B, Lires-Dean M, Fuentes-Boquete I, Arenas J, Blanco FJ (2006). Mitochondrial activity is modulated by TNFalpha and IL-1beta in normal human chondrocyte cells. Osteoarth Cartil.

[85] Ye Z, Chen Y, Zhang R, Dai H, Zeng C, Zeng H, Feng H, Du G, Fang H, Cai D (2014). c-Jun N-terminal kinase - c-Jun pathway transactivates Bim to promote osteoarthritis. Can J Physiol Pharmacol.

[86] Séguin CA, Bernier SM (2003). TNFalpha suppresses link protein and type II collagen expression in chondrocytes: role of MEK1/2 and NF-kappaB signaling pathways. J Cell Physiol.

[87] Lu YC, Jayakumar T, Duann YF, Chou YC, Hsieh CY, Yu SY, Sheu JR, Hsiao G (2011). Chondroprotective role of sesamol by inhibiting MMPs expression via retaining NF-κB signaling in activated SW1353 cells. J Agric Food Chem.

[88] Halade GV, El Jamali A, Williams PJ, Fajardo RJ, Fernandes G (2011). Obesity-mediated inflammatory microenvironment stimulates osteoclastogenesis and bone loss in mice. Exp Gerontol.

[89] Park HS, Park JY, Yu R (2005). Relationship of obesity and visceral adiposity with serum concentrations of CRP, TNF-alpha and IL-6. Diabetes Res Clin Pract.

[90] Wellen KE, Hotamisligil GS (2005). Inflammation, stress, and diabetes. J Clin Investig.

[91] Gnacińska M, Małgorzewicz S, Stojek M, Łysiak-Szydłowska W, Sworczak K (2009). Role of adipokines in complications related to obesity: a review. Adv Med Sci.

[92] Xie C, Chen Q (2019). Adipokines: new therapeutic target for osteoarthritis?. Curr Rheumatol Rep.

[93] Hayes AJ, Huang WQ, Mallah J, Yang D, Lippman ME, Li LY (1999). Angiopoietin-1 and its receptor Tie-2 participate in the regulation of capillary-like tubule formation and survival of endothelial cells. Microvasc Res.

[94] Polverini PJ (2002). Angiogenesis in health and disease: insights into basic mechanisms and therapeutic opportunities. J Dent Educ.

[95] Lambert C, Mathy-Hartert M, Dubuc JE, Montell E, Vergés J, Munaut C, Noël A, Henrotin Y (2012). Characterization of synovial angiogenesis in osteoarthritis patients and its modulation by chondroitin sulfate. Arthritis Res Ther.

[96] Kasama T, Isozaki T, Odai T, Matsunawa M, Wakabayashi K, Takeuchi HT, Matsukura S, Adachi M, Tezuka M, Kobayashi K (2007). Expression of angiopoietin-1 in osteoblasts and its inhibition by tumor necrosis factor-alpha and interferon-gamma. Trans Res: J Lab Clin Med.

[97] Bacon K, Baggiolini M, Broxmeyer H, Horuk R, Lindley I, Mantovani A, Matsushima K, Murphy P, Nomiyama H, Oppenheim J, Rot A (2001). Chemokine/chemokine receptor nomenclature. J Leukoc Biol.

[98] Balabanian K, Lagane B, Infantino S, Chow KY, Harriague J, Moepps B, Arenzana-Seisdedos F, Thelen M, Bachelerie F (2005). The chemokine SDF-1/CXCL12 binds to and signals through the orphan receptor RDC1 in T lymphocytes. J Biol Chem.

[99] Li J, Chen H, Zhang D, Xie J, Zhou X (2021). The role of stromal cell-derived factor 1 on cartilage development and disease. Osteoarth Cartil.

[100] Kim GW, Han MS, Park HR, Lee EJ, Jung YK, Usmani SE, Ulici V, Han SW, Beier F (2015). CXC chemokine ligand 12a enhances chondrocyte proliferation and maturation during endochondral bone formation. Osteoarth Cartil.

[101] Dai Y, Liu S, Xie X, Ding M, Zhou Q, Zhou X (2019). MicroRNA-31 promotes chondrocyte proliferation by targeting C-X-C motif chemokine ligand 12. Mol Med Rep.

[102] Zheng X, Zhao FC, Pang Y, Li DY, Yao SC, Sun SS, Guo KJ (2017). Downregulation of miR-221-3p contributes to IL-1β-induced cartilage degradation by directly targeting the SDF1/CXCR4 signaling pathway. J Mol Med.

[103] Xu Q, Sun XC, Shang XP, Jiang HS (2012). Association of CXCL12 levels in synovial fluid with the radiographic severity of knee osteoarthritis. J Invest Med: Off Publ Am Federation for Clinical Research.

[104] Kanbe K, Takagishi K, Chen Q (2002). Stimulation of matrix metalloprotease 3 release from human chondrocytes by the interaction of stromal cell-derived factor 1 and CXC chemokine receptor 4. Arthritis Rheum.

[105] Jones SW, Brockbank SM, Mobbs ML, Le Good NJ, Soma-Haddrick S, Heuze AJ, Langham CJ, Timms D, Newham P, Needham MR (2006). The orphan G-protein coupled receptor RDC1: evidence for a role in chondrocyte hypertrophy and articular cartilage matrix turnover. Osteoarth Cartil.

[106] Lu W, He Z, Shi J, Wang Z, Wu W, Liu J, Kang H, Li F, Liang S (2019). AMD3100 attenuates post-traumatic osteoarthritis by maintaining transforming growth factor-β1-induced expression of tissue inhibitor of metalloproteinase-3 via the phosphatidylinositol 3-kinase/akt pathway. Front Pharmacol.

[107] Lu W, Shi J, Zhang J, Lv Z, Guo F, Huang H, Zhu W, Chen A (2016). CXCL12/CXCR4 axis regulates aggrecanase activation and cartilage degradation in a post-traumatic osteoarthritis rat model. Int J Mol Sci.

[108] Lisignoli G, Toneguzzi S, Piacentini A, Cristino S, Grassi F, Cavallo C, Facchini A (2006). CXCL12 (SDF-1) and CXCL13 (BCA-1) chemokines significantly induce proliferation and collagen type I expression in osteoblasts from osteoarthritis patients. J Cell Physiol.

[109] Chen Y, Lin S, Sun Y, Guo J, Lu Y, Suen CW, Zhang J, Zha Z, Ho KW, Pan X (2017). Attenuation of subchondral bone abnormal changes in osteoarthritis by inhibition of SDF-1 signaling. Osteoarth Cartil.

[110] Qin H, Zhao X, Hu YJ, Wang S, Ma Y, He S, Shen K, Wan H, Cui Z, Yu B (2021). Inhibition of SDF-1/CXCR4 axis to alleviate abnormal bone formation and angiogenesis could improve the subchondral bone microenvironment in osteoarthritis. Biomed Res Int.

[111] Martin AR, Patel JM, Locke RC, Eby MR, Saleh KS, Davidson MD, Sennett ML, Zlotnick HM, Chang AH, Carey JL (2021). Nanofibrous hyaluronic acid scaffolds delivering TGF-β3 and SDF-1α for articular cartilage repair in a large animal model. Acta Biomater.

[112] Tu M, Yao Y, Qiao FH, Wang L (2019). The pathogenic role of connective tissue growth factor in osteoarthritis. Biosci Rep.

[113] Baxter RC, Twigg SM (2009). Actions of IGF binding proteins and related proteins in adipose tissue. Trends Endocrinol Metab.

[114] Bazzazi H, Zhang Y, Jafarnejad M, Isenberg JS, Annex BH, Popel AS (2018). Computer simulation of TSP1 inhibition of VEGF-Akt-eNOS: an angiogenesis triple threat. Front Physiol.

[115] Lu A, Miao M, Schoeb TR, Agarwal A, Murphy-Ullrich JE (2011). Blockade of TSP1-dependent TGF-β activity reduces renal injury and proteinuria in a murine model of diabetic nephropathy. Am J Pathol.

[116] Omoto S, Nishida K, Yamaai Y, Shibahara M, Nishida T, Doi T, Asahara H, Nakanishi T, Inoue H, Takigawa M (2004). Expression and localization of connective tissue growth factor (CTGF/Hcs24/CCN2) in osteoarthritic cartilage. Osteoarth Cartil.

[117] Blaney Davidson EN, Vitters EL, Mooren FM, Oliver N, Berg WB, van der Kraan PM (2006). Connective tissue growth factor/CCN2 overexpression in mouse synovial lining results in transient fibrosis and cartilage damage. Arthritis Rheum.

[118] Farrell B, Breeze AL (2018). Structure, activation and dysregulation of fibroblast growth factor receptor kinases: perspectives for clinical targeting. Biochem Soc Trans.

[119] Im HJ, Muddasani P, Natarajan V, Schmid TM, Block JA, Davis F, van Wijnen AJ, Loeser RF (2007). Basic fibroblast growth factor stimulates matrix metalloproteinase-13 via the molecular cross-talk between the mitogen-activated protein kinases and protein kinase Cdelta pathways in human adult articular chondrocytes. J Biol Chem.

[120] Yan D, Chen D, Cool SM, van Wijnen AJ, Mikecz K, Murphy G, Im HJ (2011). Fibroblast growth factor receptor 1 is principally responsible for fibroblast growth factor 2-induced catabolic activities in human articular chondrocytes. Arthritis Res Ther.

[121] Weng T, Yi L, Huang J, Luo F, Wen X, Du X, Chen Q, Deng C, Chen D, Chen L (2012). Genetic inhibition of fibroblast growth factor receptor 1 in knee cartilage attenuates the degeneration of articular cartilage in adult mice. Arthritis Rheum.

[122] Tang J, Su N, Zhou S, Xie Y, Huang J, Wen X, Wang Z, Wang Q, Xu W, Du X (2016). Fibroblast growth factor receptor 3 inhibits osteoarthritis progression in the knee joints of adult mice. Arthritis Rheumatol.

[123] Zhou S, Xie Y, Li W, Huang J, Wang Z, Tang J, Xu W, Sun X, Tan Q, Huang S (2016). Conditional deletion of Fgfr3 in chondrocytes leads to osteoarthritis-like defects in temporomandibular joint of adult mice. Sci Rep.

[124] Kuang L, Wu J, Su N, Qi H, Chen H, Zhou S, Xiong Y, Du X, Tan Q, Yang J (2020). FGFR3 deficiency enhances CXCL12-dependent chemotaxis of macrophages via upregulating CXCR7 and aggravates joint destruction in mice. Ann Rheum Dis.

[125] Xie Y, Su N, Yang J, Tan Q, Huang S, Jin M, Ni Z, Zhang B, Zhang D, Luo F (2020). FGF/FGFR signaling in health and disease. Signal Transduct Target Ther.

[126] Xie Y, Zinkle A, Chen L, Mohammadi M (2020). Fibroblast growth factor signalling in osteoarthritis and cartilage repair. Nat Rev Rheumatol.

[127] Nagashima M, Hasegawa J, Kato K, Yamazaki J, Nishigai K, Ishiwata T, Asano G, Yoshino S (2001). Hepatocyte growth factor (HGF), HGF activator, and c-Met in synovial tissues in rheumatoid arthritis and osteoarthritis. J Rheumatol.

[128] Nakamura T, Nawa K, Ichihara A (1984). Partial purification and characterization of hepatocyte growth factor from serum of hepatectomized rats. Biochem Biophys Res Commun.

[129] Miyazawa K, Shimomura T, Kitamura N (1996). Activation of hepatocyte growth factor in the injured tissues is mediated by hepatocyte growth factor activator. J Biol Chem.

[130] Gherardi E, Gray J, Stoker M, Perryman M, Furlong R (1989). Purification of scatter factor, a fibroblast-derived basic protein that modulates epithelial interactions and movement. Proc Natl Acad Sci USA.

[131] Mabey T, Honsawek S, Saetan N, Poovorawan Y, Tanavalee A, Yuktanandana P (2014). Angiogenic cytokine expression profiles in plasma and synovial fluid of primary knee osteoarthritis. Int Orthop.

[132] Catizone A, Ricci G, Tufano MA, Perfetto B, Canipari R, Galdieri M (2010). Hepatocyte growth factor (HGF) modulates Leydig cell extracellular matrix components. J Androl.

[133] Que W, Liu H, Yang Q (2022). CircPRKCH modulates extracellular matrix formation and metabolism by regulating the miR-145/HGF axis in osteoarthritis. Arthritis Res Ther.

[134] Huang Y, He B, Wang L, Yuan B, Shu H, Zhang F, Sun L (2020). Bone marrow mesenchymal stem cell-derived exosomes promote rotator cuff tendon-bone healing by promoting angiogenesis and regulating M1 macrophages in rats. Stem Cell Res Ther.

[135] Noh KC, Park SH, Yang CJ, Lee GW, Kim MK, Kang YH (2018). Involvement of synovial matrix degradation and angiogenesis in oxidative stress-exposed degenerative rotator cuff tears with osteoarthritis. J Shoulder Elbow Surg.

